# Functional Outcomes and Efficiency of Rehabilitation in a National Cohort of Patients with Guillain - Barré Syndrome and Other Inflammatory Polyneuropathies

**DOI:** 10.1371/journal.pone.0110532

**Published:** 2014-11-17

**Authors:** Roxana Alexandrescu, Richard John Siegert, Lynne Turner-Stokes

**Affiliations:** 1 Department of Palliative Care, Policy and Rehabilitation, School of Medicine, King’s College London, London, United Kingdom; 2 School of Public Health and Psychosocial Studies and School of Rehabilitation and Occupational Studies, Auckland University of Technology, Auckland, New Zealand; 3 Regional Rehabilitation Unit, Northwick Park Hospital, London, United Kingdom; Hannover Medical School, Germany

## Abstract

**Objectives:**

To describe functional outcomes, care needs and cost-efficiency of hospital rehabilitation for a UK cohort of inpatients with complex rehabilitation needs arising from inflammatory polyneuropathies.

**Subjects and Setting:**

186 patients consecutively admitted to specialist neurorehabilitation centres in England with Guillain-Barré Syndrome (n = 118 (63.4%)) or other inflammatory polyneuropathies, including chronic inflammatory demyelinating polyneuropathy (n = 15 (8.1%) or critical illness neuropathy (n = 32 (17.2%)).

**Methods:**

Cohort analysis of data from the UK Rehabilitation Outcomes Collaborative national clinical dataset. Outcome measures include the UK Functional Assessment Measure, Northwick Park Dependency Score (NPDS) and Care Needs Assessment (NPCNA). Patients were analysed in three groups of dependency based on their admission NPDS score: ‘low’ (NPDS<10), ‘medium’ (NPDS 10–24) and ‘high’ (NPDS ≥25). Cost-efficiency was measured as the time taken to offset the cost of rehabilitation by savings in NPCNA-estimated costs of on-going care in the community.

**Results:**

The mean rehabilitation length of stay was 72.2 (sd = 66.6) days. Significant differences were seen between the diagnostic groups on admission, but all showed significant improvements between admission and discharge, in both motor and cognitive function (p<0.0001). Patients who were highly dependent on admission had the longest lengths of stay (mean 97.0 (SD 79.0) days), but also showed the greatest reduction in on-going care costs (£1049 per week (SD £994)), so that overall they were the most cost-efficient to treat.

**Conclusions:**

Patients with polyneuropathies have both physical and cognitive disabilities that are amenable to change with rehabilitation, resulting in significant reduction in on-going care-costs, especially for highly dependent patients.

## Introduction

Guillain-Barré Syndrome (GBS) and other inflammatory polyneuropathies are a group of disorders that are often associated with significant long-term disability [1], [2]. In addition to motor deficits, many patients have cognitive psychosocial problems resulting in complex disability, which may sometimes require treatment in a specialist rehabilitation service [3]. However, in comparison to other long term neurological conditions (such as brain injury, stroke, or multiple sclerosis) there are relatively few published analyses of outcome in this context.

Inflammatory polyneuropathies are a clinically and pathophysiologically heterogeneous group. GBS is an acute, autoimmune condition, with a natural course fast with high disability and usually episodic immune treatment; Chronic inflammatory demyelinating polyneuropathy (CIDP) has a chronic, autoimmune, natural course, slow with ongoing disability, usually maintenance immune treatment; Critical Illness Neuropathy (CIN) is more a myopathy than a neuropathy; it is an acute, ischemic/degenerative/inflammatory disorder, associated with a prolonged period of critical illness – usually in intensive care settings. It has no immune treatment option. Its natural course natural course represents resting disability with slow recovery and cognitive deficits due to the systemic inflammatory/infectious etiology. Given this heterogeneity, differences in outcome are expected between these groups.

The existing literature tends to focus on GBS, where a number of studies have explored outcomes relating to predominantly physical disability (eg using the Functional Independence Measure (FIM)) or quality of life measures [4]–[11]. Few studies have focused on the cognitive psychosocial functional deficits of this group, and none have so far addressed issues relating to cost-effectiveness, or which patients may be the most cost-efficient to treat.

One of the key challenges of healthcare services across the world is to identify those services that are not only effective, but also represent value for money. Porter and Teisberg [12] introduced the concept of value-based health care (VBHC) where the goal is not to minimize costs but to maximize “value,” defined as ‘patient outcomes divided by costs’. In the context of routine clinical practice, direct costing data are not always available and a number of proxies have been introduced as indices of cost-efficiency. In rehabilitation, the FIM-efficiency index (FIM gain ÷ length of stay) has been used in some countries as a proxy for cost-efficiency [13], [14] on the basis that functional gain is correlated with reduced on-going care costs and length of stay in rehabilitation is a key determinator of treatment costs. However, such estimations are frequently confounded by floor and ceiling effects in the index measure. This is particularly a problem with the FIM, which is heavily focussed on physical disability, and offers scant coverage of cognitive or psychosocial needs.

In England, the UK Rehabilitation Outcomes Collaborative (UKROC) database collates episode data for inpatients admitted to specialist rehabilitation services. In addition to providing the commissioning dataset, it also routinely provides national benchmarking on quality, outcomes and cost efficiency of rehabilitation. Within the dataset, functional gain is evaluated using the UK Functional Assessment Measure (UK FIM+FAM) [15], [16], which extends the motor-dominated FIM to provide a more rounded assessment of cognitive and psychosocial function. Cost-efficiency is computed in terms of the length of time taken to offset the initial costs of rehabilitation through savings in the on-going costs of community care, as estimated by the Northwick Park Care Needs Assessment [17]. Previously published analyses using these indices have demonstrated the cost efficiency of rehabilitation for patient with highly complex needs who are often denied rehabilitation in other healthcare systems on the basis that they would not be expected to make significant gains on the FIM alone [18].

The aim of this paper was to validate the factor structure of the UK FIM+FAM within the study population and to describe functional outcomes, including change care needs and cost-efficiency following specialist rehabilitation for patients with complex disability arising from inflammatory polyneuropathies. We also examined care needs and outcome across the different diagnostic groups and compared outcomes and co-efficiency of rehabilitation for patients with different levels of dependency.

## Methods

### Setting

Rehabilitation Services in England are classified into three different levels. Level 1 (tertiary or regional services) and Level 2 (district or supra-district) specialist rehabilitation services take a selected population of patients with complex needs for rehabilitation that are beyond the scope of their local (level 3) general rehabilitation services [3].

### Data source

The UKROC database collates the national clinical dataset for in-patient specialist (Levels 1 and 2) rehabilitation in England. The dataset comprises socio-demographic and process data (waiting times, discharge destination etc) as well as clinical information on rehabilitation needs, inputs and outcomes. Full details may be found on the UKROC website http://www.csi.kcl.ac.uk/ukroc.html. Data collection is now a mandatory requirement for commissioning of specialist rehabilitation services in England, but between 2010 and 2013 it was voluntary and contributing centres could report one of three measures, the Barthel Index, the FIM or UK FIM+FAM. Between 1.1.10 and 30.4.13, a total of 319 inpatient episodes were recorded for patients admitted with a diagnosis of Guillain-Barre Syndrome or other inflammatory polyneuropathy to a total of 56 specialist rehabilitation units. Of these, 217 were admitted to services that record the UK FIM+FAM and had complete UK FIM+FAM admission scoring. Of these 186 patients (86%, 186/217) had complete UK FIM+FAM scores at both admission and discharge and were taken as the study sample extracted for this analysis. This sample included data from 45 specialist rehabilitation units, representing approximately 75% of the total number of specialised inpatient neurorehabilitation centres (level 1 and 2) within England during the study period.

### Measurements

The FIM+FAM is a global measure of disability that includes the 18-item FIM (version 4) and adds a further 12 items, mainly addressing psychosocial function so that the total FIM+FAM comprises 30 items (16 motor items and 14 cognitive items). Each item is scored on a seven-point ordinal scale from 1 (total dependence) to 7 (complete independence). Originally developed in the US in the 1990 s [19] the UK FIM+FAM was published in 1999 and now forms the principal measure of inpatient rehabilitation programme outcomes in the UKROC database [15], [16]. There are also centres using it in Europe, South America and Australasia.

A separate optional 6-item Extended Activities of Daily Living (EADL) module has been developed with the aim to extend the upper range of the tool [20]. It represents basic household tasks that contribute to independence after the patient has been discharged. The UK FIM+FAM is completed within 10 days of admission and within the last week before discharge to allow an assessment of the patient’s functional gains made during the episode of care.

The Northwick Park Dependency Score (NPDS) is an ordinal scale of dependency on nursing time (number of helpers and time taken to assist with each task) designed to assess needs for care and nursing in clinical rehabilitation settings [21]. It is divided into two sections.

- Basic Care Needs (BCN) (16 ordinal items with score ranges varying from 0 to 3–5, higher scores indicating a greater level of dependency – ie the opposite direction to the FIM+FAM)
- Special Nursing Needs (SNN) (7 items scored as dichotomous variables, with a score of either 0 (absent) or 5 (present)).

The NPDS is shown to be a valid and reliable measure of needs for care and nursing in rehabilitation settings [22]. It supports categorisation of patients into three dependency groups based on their admission NPDS scores:

- Low dependency (NPDS <10): patients are largely independent for basic self care,
- Medium (NPDS 10–24): patients generally require help from one person for most self-care tasks
- High (NPDS ≥25): patients require help from two or more persons for most care tasks and often also have special nursing needs.

The NPDS also translates via a computerised algorithm to Northwick Park Care Needs Assessment (NPCNA) [23] which provides a daily timetable of care needs, and also estimates the total ‘care hours per week’ (RCH) and the approximate weekly cost of care (£/week) in the community, based on the UK care agency costs. The NPCNA provides a generic assessment of care needs, regardless of who provides and pays for them. The estimated cost of care is therefore independent of individual circumstances or local policy for the provision continuing care, which varies widely across the UK.

Although there is no formal accreditation process for use of the UK FIM+FAM and NPDS, the attendance of UK FIM+FAM training by at least a core team of staff is requirement for UKROC registration. All units that are registered with UKROC have access to free training and updates in workshops that are run several times a year to keep staff up to date, and telephone support is also provided by the UKROC team.

### Cost Efficiency of rehabilitation

Within the UKROC dataset, the cost efficiency is calculated as the time taken to offset the cost of rehabilitation by the resulting savings in the cost of on-going care in the community. This is calculated from ‘Mean episode cost of rehabilitation’ divided by ‘mean reduction in weekly cost of care’ from admission to discharge, as estimated by the NPCNA. The cost of episode was calculated per patient as bed-day cost multiplied by length of stay in days. In this study, the cost per bed-day was calculated retrospectively based on the same costing methodology as our previously published cost analysis [24]: The mean per diem costs were calculated as £551.2 for tertiary (level 1) services contributing to this sample and £418.1 for the specialist (level 2) rehabilitation units. The analysis of cost-efficiency was further restricted to the cohort of patients that had relevant NPDS and cost information available (N = 102).

### Data analysis

As the dataset was of reasonable size and the data near normally distributed, parametric techniques were used and statistical analysis was carried out using IBM SPSS statistics v21. Although the UK FIM+FAM has been validated using UKROC data for a general neurorehabilitation sample^16^, because it was not previously validated specifically in this population, we carried out a preliminary principal component analysis (PCA) to confirm the overall factor structure. We used admission and discharge scores data pooled together, N = 368 to extend across the scoring range and to allow for a minimum sample size of 10 cases per item. To assess internal reliability, we calculated Cronbach’s coefficient-alpha for the whole scale and subscales as determined by the PCA. A coefficient-alpha between 0.70 and 0.95 is considered to reflect good internal consistency. The factor structure was then tested using confirmatory factor analysis in IBM SPSS AMOS 21. Goodness of fit was assessed with four indices: root-mean-square error of approximation RMSEA, comparative fit index/Tucker-Lewis index CFI/TLI and goodness of fit index GFI. Due to the sample size, the chi-square difference test was not considered a relevant fit index.

Paired T tests were used to compare differences in scores between admission and discharge. To identify differences between groups, we used one-way ANOVA with Bonferroni correction. For all statistical tests, two-tailed p values of <0.05 were considered to be statistically significant. Chi-squared test was used for categorical data. ‘FIM+FAM splats’ (radar charts) were employed to show item-by-item changes between mean scores at admission and discharge.

### Ethics

The UKROC database collates de-identified data as part of routine clinical practice and the programme registered as a Payment by Results Improvement Project. The analysis of this routinely-collected data is classed as service evaluation, which does not require research ethics permission in the UK.

## Results

Demographics of the UKROC data and study population are shown in Table 1. We compare the study sample data (N = 186) with the cohort representing patients with complete UK FIM+FAM scoring at admission (N = 217) and the total cohort of patients with polyneuropathies within the UKROC database (N = 319). No significant differences were seen in either the socio-demographic characteristics or the proportionate representation of the different diagnostic groups.

**Table 1:** Socio-demographic characteristics of the study population.

	UKROC sample*(N = 319)	Admission sample **(N = 217)	Study sample***(N = 186)
Age, years, mean (SD)	55.0 (16.3)	54.0 (16.2)	53.6 (16.2)
Length of stay, days, mean (SD)	68.9 (66.9)	73.5 (67.7)	72.2 (66.6)
Time since onset, months, mean (SD)Male, n (%)	5.8 (24.7)190 (59.6)	4.3 (13.3)134 (61.8)	4.5 (14.2)116 (62.4)
Diagnosis, n (%)			
Guillain Barre Syndrome	193 (60.5)	139 (64.1)	118 (63.4)
CIDP	31 (9.7)	16 (7.4)	15 (8.1)
CIN	59 (18.5)	39 (18.0)	32 (17.2)
Unspecified neuropathies	37 (11.6)	23 (10.6)	21 (11.0)

CIDP: chronic inflammatory demyelinating polyneuropathy; CIN: critical illness neuropathy;

*The UKROC data extract comprised the 319 patients extracted from the UKROC database with a diagnosis of polyneuropathy; **Admission sample comprised 217 cases that had complete UK FIM+FAM scores on admission; ***The study sample comprised the 186 cases that had complete UK FIM+FAM scores on both admission and discharge.

The study sample comprised 116 male and 70 female patients with a mean age at admission of 53.6 (sd = 16.2) years, range 18–85 years. The mean rehabilitation length of stay was 72.2 (sd = 66.6) days. There were 118 (63.4%) patients with Guillain-Barré Syndrome (GBS), 15 (8.1%) patients with CIDP and 32 (17.2%) patients with CIN and myopathy. A further 37 (11.1%) had unspecified neuropathies.

The principal components analysis revealed four components with Eigenvalues over 1 (suggesting four sources of variance, overall 69.6% of the variance). Most of the items loaded strongly on the first principal component (26 out of 30 items above 0.40). Based on the previous research^16^ we rotated, using Varimax procedure, two main factors, representing the motor and cognitive dimensions (accounting for 45.9% and respectively 14.5% of the variance). Four items (‘Swallowing’, ‘Writing’, ‘Leisure activities’, and ‘Safety awareness’) which loaded significantly (>0.3) onto both factors, were assigned on the basis of best clinical fit (Table 2). The Cronbach’s alpha was 0.97 and 0.89 for the motor and cognitive domains and 0.96 for the entire scale. The reliability of the hypothesized two-factor model was assessed by confirmatory factor analysis. Inspection of the modification indices suggested model fit would be significantly improved if items ‘Writing’ and ‘Leisure activities’, were allowed to load on both the Motor and Cognitive factors. Modification indices related to the covariance of the pairing of errors of the some of the items had also large values (Eating and Swallowing; Grooming and Dressing upper; Transfer bed and Transfer toilet; Stairs and Mobility). This might have been triggered by a degree of overlap in item content. The model fit has been further improved by adding covariance between these pairs of item error terms. For the final model the RMSEA was 0.105, CFI/TLI 0.851/0.838 and the GFI was 0.719. The final model supported the two-factor hypothesized structure of the scale and the previously published factor structure [16].

**Table 2:** Principal components analysis with two - factor varimax rotation of the UK FIM FAM (N = 368).

Item	Mean (SD)	Single factor1st PC	Two factor	
			Motor	Cognitive
Eating	5.6 (2.0)	.53	.69	
Swallowing	6.5 (1.4)	.21	.30	(.34)
Grooming	5.2 (2.0)	.67	.80	
Bathing	4.2 (2.1)	.84	.90	
Dressing upper	4.0 (2.3)	.76	.85	
Dressing lower	4.9 (2.2)	.83	.90	
toileting	4.3 (2.4)	.84	.91	
Bladder	4.9 (2.4)	.65	.77	
Bowel	4.9 (2.4)	.68	.79	
Transfer bed	4.4 (2.3)	.88	.92	
Transfer toilet	4.2 (2.3)	.87	.92	
Transfer bath	3.8 (2.4)	.81	.88	
Transfer car	3.4 (2.5)	.71	.83	
Locomotion	4.2 (2.2)	.65	.78	
Stairs	2.6 (2.3)	.61	.77	
Mobility	2.8 (2.1)	.54	.72	
Comprehension	6.8 (0.8)	.54		.73
Expression	6.7 (0.9)	.58		.75
Reading	6.4 (1.6)	.40		.59
Writing	5.3 (2.3)	.41	(.53)	.35
Speech	6.8 (0.8)	.28		.52
Social interaction	6.6 (1.0)	.62		.77
Emotional status	6.0 (1.6)	.32		.55
Adjustment	5.9 (1.4)	.53		.67
Leisure activities	5.2 (1.8)	.46	(.52)	.43
Problem solving	6.2 (1.6)	.69		.80
Memory	6.5 (1.3)	.67		.81
Orientation	6.5 (1.1)	.50		.70
Concentration	6.8 (0.9)	.68		.81
Safety awareness	6.0 (1.6)	.38	(.33)	.52

All factor loadings rounded to two decimal points. Loadings 0.20 removed for clarity.

PC, principal component.

**Figure 1:**
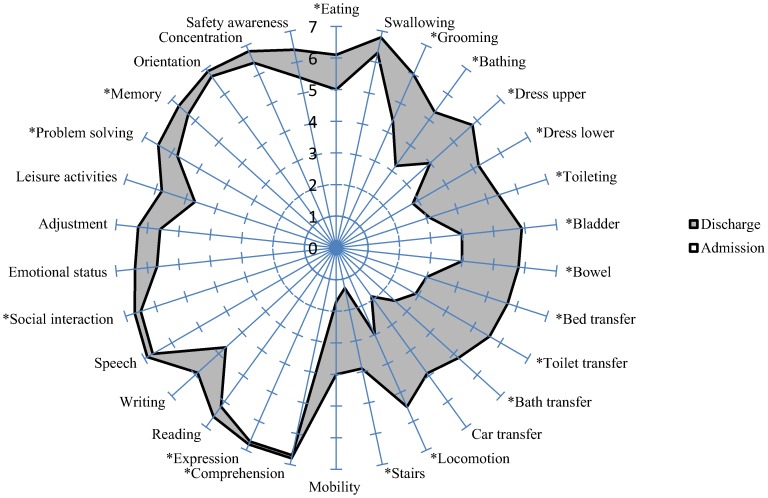
The UK FIM+FAM: UK Functional Independence Measure and Assessment Measure; (*FIM Items are explicitly shown). The items are displayed as ‘spokes of the wheel’ by level (1 (total dependency) to 7 (total independency)) from the centre outwards. The shaded area represents the gain in item mean scores from admission to discharge.

Figure 1 shows a composite UK FIM+FAM-splat for the study sample. The shaded area represents the change in mean value from admission and discharge for each of the 30 items. The graphical representation provides a clear picture of the areas with the greatest improvement. As expected the largest gains were seen in the motor items, in particular items related to mobility (transfers, locomotion, stairs), continence and self care (bathing, dressing and toileting). Significant gains were also seen within the cognitive/psychosocial items (especially writing, leisure activities and adjustment to limitations).

Table 3 summarises the overall changes in functional independence and care needs between admission and discharge for the whole study sample. Significant changes were seen for all parameters. The mean gain in UK FIM+FAM from admission to discharge was 33.8 (95%CI, 30.6 to 37.0) for the motor scale and 7.3 (95%CI, 5.7 to 8.8) for the cognitive scale consistent with the higher starting points for these items. Mean scores on admission for the EADL items were substantially lower than for the standard FIM+FAM items, but gains of 1–2 points per item resulted an overall gain score for 7.6 (95%CI, 6.2 to 9.1).

**Table 3:** The UK FIM+FAM and EADL item means on admission and discharge, and change scores (N = 186).

Item (scale)	Admission Mean (SD)	Discharge Mean (SD)	Mean difference	95% CI		P value
**FIM scores (n = 186)**						
FIM motor	43.4 (21.1)	71.2 (20.9)	−27.8	−30.6	−25.1	<0.0001
FIM cognitive	31.9 (4.9)	33.5 (4.0)	−1.5	−2.0	−1.0	<0.0001
FIM total	75.2 (23.2)	104.9 (22.9)	−29.7	−32.6	−26.7	<0.0001
**FIM+FAM scores (n = 186)**						
FIM+FAM motor	53.2 (23.5)	87.0 (24.8)	−33.8	−37.0	−30.6	<0.0001
FIM+FAM cognitive	84.1 (13.5)	91.4 (10.7)	−7.3	−8.8	−5.7	<0.0001
FIM+FAM total	137.3 (32.3)	178.4 (32.4)	−41.1	−45.2	−36.9	<0.0001
**EADL scores (n = 84)**						
Meal preparation	1.9 (1.6)	4.1 (2.1)	−2.2	−2.5	−1.8	<0.0001
Laundry	1.7 (1.4)	3.1 (2.3)	−1.4	−1.8	−1.0	<0.0001
Housework	1.5 (1.2)	2.8 (2.1)	−1.3	−1.6	−0.9	<0.0001
Shopping	1.6 (1.2)	3.1 (2.1)	−1.5	−1.9	−1.2	<0.0001
Financial	3.5 (2.7)	4.8 (2.7)	−1.3	−1.7	−0.9	<0.0001
EADL total	10.3 (6.8)	17.9 (9.4)	−7.6	−9.1	−6.2	<0.0001
**NPDS (n = 102)**						
Basic Care Needs	20.9 (11.9)	9.4 (10.8)	11.5	9.5	13.4	<0.0001
Special Nursing Needs	5.0 (5.9)	2.8 (4.7)	2.2	1.2	3.3	<0.0001
Total NPDS	25.9 (15.8)	12.3 (14.3)	13.7	11.1	16.2	<0.0001
**NPCNA (n = 102) – estimated care needs in the community**						
Care hours per week	42.9 (19.9)	20.9 (18.8)	22.0	18.5	25.5	<0.0001
Weekly care costs £	1321.7 (99.0)	601.5(776.1)	720.3	545.2	895.3	<0.0001

UK FIM+FAM: The UK Functional Assessment Measure; EADL: The Extended Activities of Daily Living; NPDS: Northwick Park Dependency Score; NPCNA: Northwick Park Care Needs Assessment.

The mean reduction in overall dependency (total NPDS score) was 13.7 ((%%CI 11.1–16.2), of which the majority of change was in the Basic Care Needs section (11.5 (95%CI, 9.5 to 13.4)). This translates into a reduction of 37.4 care hours per week with mean cost savings £720.3 (SD £891.3) per week.

Table 4 summarises the cost-efficiency data. The overall mean cost of the in-patient rehabilitation episode was £34,714 (SD £34,338), so that the time taken to offset the initial investment in rehabilitation by saving in on-going care costs was 12 months. When analysed in different groups of dependency, as expected the length of stay (and therefore cost of rehabilitation) was greatest in the high dependency group - £46,435 compared with £26,549 (medium) and £17,005 (low) dependency groups. However, the reduction in care costs were also correspondingly greater, so that the time to offset the cost of admission was shortest for the high dependency group −11 months compared with 13 months (medium) and 22 months low. Of note, half of the patients were classified within the highest NPDS group, confirming that (at least for this part of the analysis) this was a selected group of patients with complex rehabilitation needs.

**Table 4:** Efficiency of rehabilitation by dependency and diagnosis group (N = 102).

	Dependency group			
	All*N = 102	NPDS<10 (Low)N = 19	NPDS 10–24N = 32	NPDS>25 (High)N = 51
Length of stay, days, mean (SD)	72.9 (64.7)	34.5 (19.9)	57.3 (35.0)	97.0 (79.0)
Cost of episode, mean (SD), £	34714.4 (34337.9)	17005.1 (10681.6)	26549.3 (17628.8)	46435.3 (42965.6)
Reduction in weekly care costs, mean (SD), £	720.3 (891.3)	191.4 (373.9)	510.0 (719.1)	1049.2 (994.4)
Time taken to offset the cost of rehabilitation	12.0 months	22.2 months	13.0 months	11.1 months
FIM-efficiency	0.38	0.44	0.48	0.33
	**Diagnosis group**			
	**Guillain Barre** **Syndrome N = 63**	**CIDP** **N = 7**	**CIN** **N = 20**	**Other** **N = 12**
Length of stay, days, mean (SD)	80.0 (72.7)	57.1 (26.2)	61.3 (41.3)	64.2 (67.1)
Cost of episode, mean (SD), £	38491.1 (39778.8)	25297.5 (12543.7)	28250.8 (19965.2)	31153.2 (30087.1)
Reduction in weekly care costs, mean (SD), £	781.7 (865.0)	330.0 (566.9)	948.5 (1169.6)	245.3 (281.6)
Time taken to offset the cost of rehabilitation	12.3 months	19.2 months	7.4 months	31.7 months
FIM-efficiency	0.39	0.37	0.53	0.31

NPDS: The Northwick Park Dependency Score; *102 cases had complete information on costs.

Cost of episode = bed-day cost multiplied by length of stay;

Time taken to offset cost of rehabilitation is calculated on a population basis from ‘mean episode cost ÷ mean reduction in NPCNA-estimated weekly cost of care from admission to discharge’.

FIM-efficiency is calculated on a population basis as ‘FIM gain ÷ length of stay’.

Table 5 shows comparison of the socio-demographic characteristics and rehabilitation outcomes at admission and discharge by diagnosis group. No significant differences were seen in respect of age, gender or length of stay. However, there were some statistically significant differences in respect of their function. At both admission and discharge, patients with CIN had significant lower scores for cognitive function than those with GBS or CIDP. They also had significant greater needs for special nursing, resulting in overall higher care needs and costs. Patients with GBS occupied the mid position, whilst those with CIPD where generally the least dependent – although had they been discharged directly into the community at the time of admission, their weekly care costs would still have averaged £835 per week.

**Table 5:** Socio-demographic characteristics and scales scores by diagnosis group (N = 186).

	Guillain Barre Syndrome	CIDP	CIN	p value
	N = 118	N = 15	N = 32	
Age, years, mean (SD)	53.6 (16.6)	52.8 (22.6)	53.7 (13.5)	0.998
Length of stay, days, mean (SD)	79.8 (76.0)	55.7 (38.1)	56.4 (41.1)	0.218
Male, n (%)	77 (65.3)	12 (80.0)	17 (53.1)	0.138
**Scores on admission, mean (SD)**	**N = 118**	**N = 15**	**N = 32**	
**FIM**				
FIM motor scale	42.9 (21.4)	48.6 (23.9)	38.0 (18.1)	0.253
FIM cognitive scale	32.9 (3.3)	32.1 (3.9)	28.2 (8.5)	<0.0001
FIM total	75.6 (22.5)	80.7 (24.9)	66.2 (23.4)	0.064
**UK FIM+FAM**				
FIM+FAM motor scale	50.8 (24.1)	59.9 (27.1)	47.3 (20.9)	0.237
FIM+FAM cognitive scale	85.4 (10.5)	86.7 (10.8)	76.3 (21.3)	0.002
FIM+FAM total	136.2 (30.7)	146.7 (31.7)	123.5 (36.7)	0.047
NPDS, N = 102*	N = 63	N = 7	N = 20	
Total Basic Care Needs	20.7 (11.8)	17.3 (9.8)	26.7 (11.6)	0.072
Total Special Nursing Needs	4.1 (5.1)	3.6 (2.4)	10.0 (7.9)	0.001
NPDS total	24.8 (14.7)	20.9 (10.6)	36.6 (17.3)	0.008
NPCNA, N = 102*	**N = 63**	**N = 7**	**N = 20**	
Estimated care hours per week	43.9 (21.1)	35.0 (17.6)	50.5 (16.1)	0.213
Estimated cost of care per week, £	1323.5 (998.7)	835.1 (613)	1877.3 (1054.4)	0.022
**Scores on discharge, mean (SD)**	**N = 118**	**N = 15**	**N = 32**	
**FIM**				
FIM motor scale	73.1(19.6)	67.9(22.0)	68.0(25.2)	0.365
FIM cognitive scale	34.1(2.3)	33.9(1.9)	31.0(7.8)	0.001
FIM total	107.2(20.4)	101.8(21.8)	99.0(31.0)	0.167
**UK FIM+FAM**				
FIM+FAM motor scale	89.4(22.9)	83.1(25.4)	82.9(30.3)	0.329
FIM+FAM cognitive scale	92.5(7.6)	93.3(4.6)	85.8(19.6)	0.007
FIM+FAM total	182.1(28.3)	176.5(27.5)	168.8(46.3)	0.122
NPDS, N = 102*	N = 63	N = 7	N = 20	
Total Basic Care Needs	8.1(9.1)	10.7(7.9)	13.0(14.8)	0.163
Total Special Nursing Needs	1.7(3.0)	2.1(2.7)	6.8(7.6)	<0.0001
NPDS total	9.8(10.9)	12.9(9.6)	19.9(21.2)	0.016
NPCNA, N = 102*				
Estimated care hours per week	20.2(17.4)	22.5(15.8)	25.9(23.6)	0.479
Estimated cost of care per week, £	535.0(665.3)	505.1(416.7)	1006.7(1157.1)	0.054
**Change score, mean (SD)**	**N = 118**	**N = 15**	**N = 32**	
**FIM**				
FIM motor scale	30.0(18.0)	19.3(15.6)	30.0(20.7)	0.099
FIM cognitive scale	1.2(2.0)	1.9(2.5)	2.8(7.0)	0.067
FIM total	31.6(18.8)	21.1(16.2)	32.8(24.5)	0.132
**UK FIM+FAM**				
FIM+FAM motor scale	36.9(20.6)	23.2(17.2)	35.7(25.1)	0.069
FIM+FAM cognitive scale	7.1(7.3)	6.6(7.6)	9.5(20.3)	0.525
FIM+FAM total	43.9(24.4)	29.8(19.5)	45.2(39.7)	0.161
NPDS, N = 102*	N = 63	N = 7	N = 20	
Total Basic Care Needs	−12.7(9.2)	−6.5(6.9)	−12.9(12.6)	0.29
Total Special Nursing Needs	−2.4 (4.6)	−1.4(2.4)	−3.0(8.6)	0.809
NPDS total	−15.1(11.2)	−8.0(7.8)	−16.0(20.0)	0.38
NPCNA, N = 102*				
Estimated care hours per week	−23.9(18.2)	−12.5(15.5)	−25.1(17.8)	0.25
Estimated cost of care per week, £	−781.7(865.0)	−330.0(566.9)	−948.5(1169.6)	0.318

CIDP: chronic inflammatory demyelinating polyneuropathy; CIN: critical illness neuropathy; Other includes neuropathy, polyneuropathy and myopathy; NPDS: The Northwick Park Dependency Score; NPCNA: The Northwick Park Care Needs Assessment; *102 cases had information on NPDS and NPCNA.

**Figure 2:**
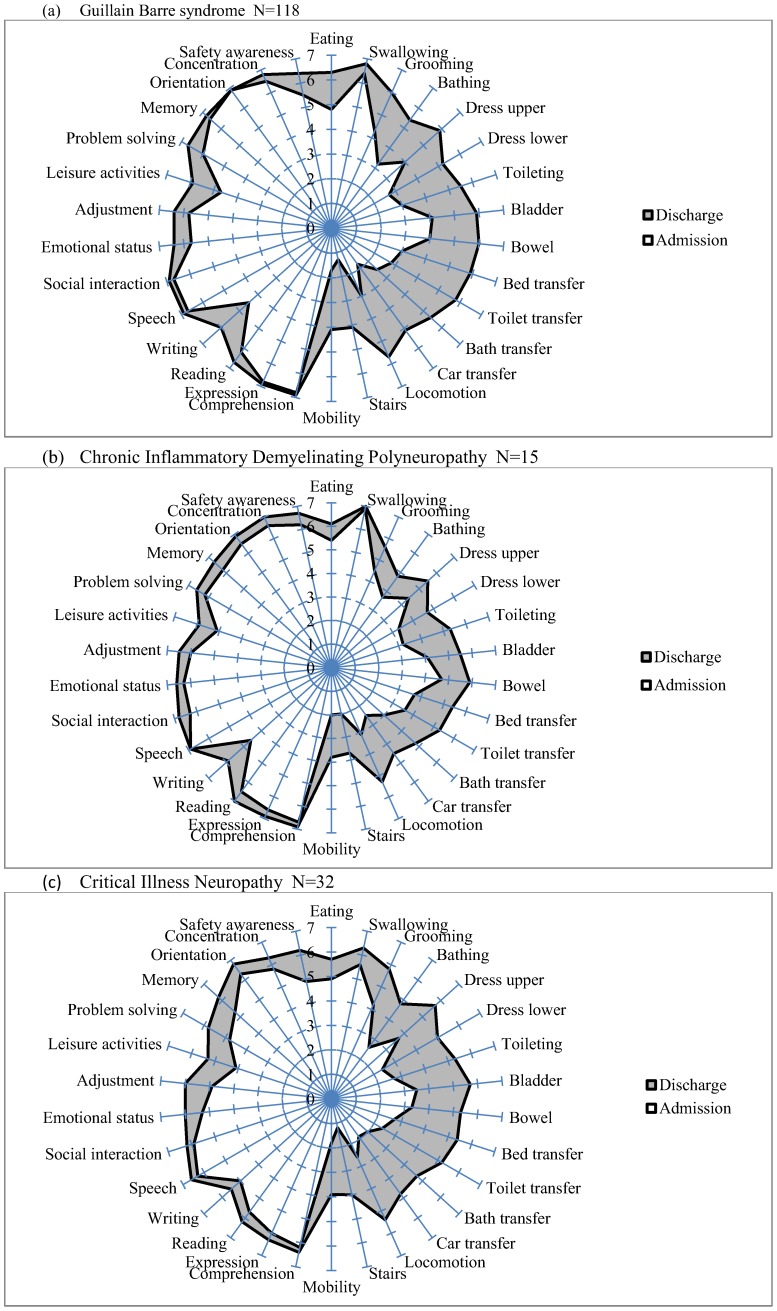
The UK FIM+FAM: UK Functional Independence Measure and Functional Assessment Measure. The items are displayed as ‘spokes of the wheel’ by level (1 (total dependency) to 7 (total independency)) from the centre outwards. The shaded area represents the gain in item mean scores from admission to discharge.

Figure 2 shows a comparison of FAM splats for the three diagnostic groups. Although CIN and GBS patients had lower scores at admission, compared with the CIDP patients they showed greater physical improvement, so that by discharge their levels of motor function were broadly similar – if not better, in the case of GBS patients. The cognitive gains were smaller with a similar pattern of the principal items affected, but it can be seen that had poorer cognitive function in CIN patients is evident broadly across the range of items within the cognitive subscale.

Comparison of cost efficiency between the diagnostic groups (Table 4) demonstrates that the GBS patients had the longest length of stay 80.0 (sd = 72.7) days and the highest episode costs (Mean £38,491 (sd = £39,779)), but the CIN patients had the greatest reduction in care costs. Consequently, despite their higher levels of dependency on admission and their cognitive disabilities, the CIN patients took the least time to offset the cost of rehabilitation (7.4 months).

## Discussion

This multi-centre analysis reports the first application of the UK FIM+FAM and Northwick Park Dependency scores to assess the functional outcome and efficiency of rehabilitation in patients with GBS and other polyneuropathies. Our findings show that, across all diagnostic and dependency subgroups, these patients make significant functional gains during rehabilitation.

As expected the majority of patients had predominantly physical disability on admission, and made substantial gains in motor function during the course of their rehabilitation programmes. Somewhat less expected was the extent of disability in cognitive/psychosocial items, which was most evident in the CIN patients, but by no means confined to this group. There is been considerable debate in the literature as to whether the FIM+FAM provides added value over the FIM as an outcome measure for complex rehabilitation. In terms of overall statistics, the FIM+FAM appears to add little benefit [25], as change in the physical domains tends to dominate the recovery pattern during post-acute rehabilitation. However at item-level, the additional psychosocial coverage offered by FIM+FAM provides a more holistic reflection of the patients’ personal goals for rehabilitation [26]. The findings here underlines the fact that, even for patients without overt cognitive impairments, acquired neurological disability can impact certain aspects of communication, cognitive and psychosocial function that may need to be actively addressed during the rehabilitation programme. In addition, patients who have been critically ill for a period often have significant cognitive disability that may go undetected unless specifically looked for.

The EADL module of the UK FIM+FAM is a relative recent addition (published in 2009) that was introduced to extend the ceiling of the FIM+FAM and to address independence in activities that are important for successful community re-integration [20]. It is included as an optional scale within the UKROC database, and so was not recorded in all patients. Few studies have so far reported outcomes for in-patient rehabilitation programmes using this scale, so it is worthy of specific mention. The low scores on admission reflect the relative difficulty of EADL tasks, in comparison with standard FIM+FAM items. In this study, gains of 1–2 points per EADL item resulted an overall gain score for 7.6. Whilst the majority of patients still required some level of assistance for most of these tasks at the time of discharge from rehabilitation, the changes observed are likely to represent clinically important change in preparation for return to the community.

Cost-efficiency of rehabilitation was evaluated in terms of the length of time taken to offset the costs if the in-patient rehabilitation programme by savings in the cost of on-going care in the community. For the sample as a whole, costs were offset within just 12 months, but the timeframes ranged from 11–22 months across the different levels of dependency. These periods are relative short compared with the figures of 16–39 months in an equivalent population of patients undergoing rehabilitation following complex acquired brain injury (ABI) [18], suggesting that in-patient rehabilitation is highly cost-efficient for this group of patients. As also seen in the ABI patients, cost efficiency was greatest in the high dependency group, despite their longer lengths of stay. FIM-efficiency, on the other hand, was greatest in the medium dependency group, which probably reflects the floor and ceiling effects of the FIM scale in patients with complex neurological disabilities.

We also interrogated the dataset for differences within the diagnostic subgroups of patients with inflammatory polyneuropathies. Patients with CIN were the most dependent and also had more severe cognitive/psychosocial disability than the other groups, both on admission and discharge. This may reflect the multiple insults that many patients in this group will have experienced in the course of prolonged stays in intensive care. However, despite their higher levels of dependency, the CIN patients took the least time to offset the cost of rehabilitation (7.4 months), confirming that specialist rehabilitation is highly cost-efficient in this group of patients.

Although the UK FIM+FAM was the primary functional outcome for this study, we also report the change in FIM scores for the purposes of comparison with other studies in the literature that have used the FIM to assess outcome form rehabilitation in patients with inflammatory peripheral neurological disorders. A few such studies exist, although most of them are single centre studies focussing patients with GBS [4]–[9], [27]–[29].

- Prasad et al reported outcomes from in-patient rehabilitation in 28 GBS patients using the FIM. They showed that activities dependent on the use of the legs (eg walking, stairs) were the most severely affected, following by activities dependent on the use of arms (eg dressing), or use of both arms and legs (eg transfers) [4]. They also showed small but significant improvements in the FIM-cognitive scale.
- Khan et al reported a good functional recovery for GBS survivors residing in the community [8]. Their median FIM scores on admission and discharge were somewhat higher than those reported here, which would be expected in comparison with our more complex group of patients who were undergoing in-patient rehabilitation in the post acute recovery stage. Of note, the median time since GBS syndrome diagnosis was 6 years in this study compared to 38 days (mean = 77.6 (sd 243.4)) in our study.
- Novak at al reported outcomes from rehabilitation for patients with CIN [29]. Mean FIM-motor scores improved from 45.6 to 69.7 at discharge, and FIM total scores changed from 78.7 to 103.3. Once again our figures were slightly lower for the group of CIN patients (mean FIM-motor scores improved from 38.0 to 68.0, and FIM-total scores from 66.2 to 98.9). The differences are likely to reflect the relative complexity of the selected population of patients presenting for specialist rehabilitation in the UK. Unfortunately Novak and colleagues did not report FIM cognitive scores, but by subtraction it would appear that their mean cognitive scores would have been approximately 29.1 on admission rising to 33.3 on discharge, in comparison with our figures of 28.2 and 30.9.

The authors recognise a number of limitations to this study:

- Although the study population represents a national cohort of patients drawn from 45 different rehabilitation units across England, and is larger than the other published studies in the literature, the sample size is still relative small, particularly for the subgroups of CIN and CIDP.
- As with any cohort design that describes functional gain in rehabilitation, spontaneous improvement during the follow-up period cannot be excluded and it might suggest that part of the functional change is not the direct result of rehabilitation
- The data were extracted from the UKROC database during a relatively early stage in its development when data reporting was not mandatory. Therefore, FIM+FAM data were available for only just over half of the total eligible population and NPDS data were available in approximately one-third. This could have led to some selection bias. From April 2013, the dataset was mandated as a requirement for commissioning of specialised rehabilitation services and routine benchmarking data on quality and cost-efficiency should come on stream from 2014, so future analyses of the dataset in 2–3 years’ time should provide a more comprehensive picture.
- The UKROC method of reporting cost-efficiency in terms of the time to offset the costs of rehabilitation is as yet specific to the UK. As noted in the methods section, the NPCNA provides only a generic estimation of care costs in the community, but it has the advantage of assessing care needs independently of how these are actually provided for. Thus it avoids variation due to local differences in provision of care and support, which are known to vary widely across the UK. It remains a problem in the UK that rehabilitation services are paid for by the healthcare commissioners, and yet savings in ongoing care costs accrue largely to social services. As yet the algorithm for estimating care hours and costs in the community has only been developed for the UK, although the NPDS has been translated into a number of different languages, and some other countries are currently exploring the development of equivalent algorithm for their local health and social care systems.
- Our methods for evaluating cost-efficiency are therefore not yet at a stage of development whether they could replace FIM-efficiency, but as this and other studies show, they have the potential to overcome some of the floor and ceiling effects that limit the usefulness of FIM-efficiency as a measure of value-based health care in rehabilitation.

In conclusion, this study builds on the existing literature provides further evidence that patients with polyneuropathies have both physical and cognitive disabilities that are amenable to change with rehabilitation. In addition, it demonstrates significant reduction in on-going care-costs, especially for highly dependent patients, and provides new evidence for the cost-efficiency of rehabilitation in this group of patients although controlled study designs are required to identify the extent to which these outcomes reflect the added value of rehabilitation over and above natural recovery. Further research is also needed to explore the underlying reason for the diagnosis-specific differences between the various sub-groups. The study also highlights the need for clinicians to be aware that, even in the absence of overt cognitive impairment, patients with inflammatory polyneuropathies may have significant cognitive, psychosocial and communicative disabilities that need to be addressed in their own right as part of the rehabilitation programme.

### Clinical messages

- Patients with polyneuropathies have both physical and cognitive disabilities that are amenable to change with rehabilitation.
- The significant reduction in on-going care-costs, especially for highly dependent patients, supports new evidence for the cost-efficiency of rehabilitation in this group of patients.
